# Identifying gene expression patterns associated with drug-specific survival in cancer patients

**DOI:** 10.1038/s41598-021-84211-y

**Published:** 2021-03-02

**Authors:** Bridget Neary, Jie Zhou, Peng Qiu

**Affiliations:** 1 School of Biological Sciences, Georgia Institute of Technology, Atlanta, GA USA; 2 Department of Biomedical Engineering, Georgia Institute of Technology and Emory University, Atlanta, GA USA

**Keywords:** Data integration, Genome informatics

## Abstract

The ability to predict the efficacy of cancer treatments is a longstanding goal of precision medicine that requires improved understanding of molecular interactions with drugs and the discovery of biomarkers of drug response. Identifying genes whose expression influences drug sensitivity can help address both of these needs, elucidating the molecular pathways involved in drug efficacy and providing potential ways to predict new patients’ response to available therapies. In this study, we integrated cancer type, drug treatment, and survival data with RNA-seq gene expression data from The Cancer Genome Atlas to identify genes and gene sets whose expression levels in patient tumor biopsies are associated with drug-specific patient survival using a log-rank test comparing survival of patients with low vs. high expression for each gene. This analysis was successful in identifying thousands of such gene–drug relationships across 20 drugs in 14 cancers, several of which have been previously implicated in the respective drug’s efficacy. We then clustered significant genes based on their expression patterns across patients and defined gene sets that are more robust predictors of patient outcome, many of which were significantly enriched for target genes of one or more transcription factors, indicating several upstream regulatory mechanisms that may be involved in drug efficacy. We identified a large number of genes and gene sets that were potentially useful as transcript-level biomarkers for predicting drug-specific patient survival outcome. Our gene sets were robust predictors of drug-specific survival and our results included both novel and previously reported findings, suggesting that the drug-specific survival marker genes reported herein warrant further investigation for insights into drug mechanisms and for validation as biomarkers to aid cancer therapy decisions.

## Introduction

Cancer has been a major focus in precision medicine because it is a heterogeneous disease with significant variations in therapeutic responses. Improved understanding of a drug’s molecular mechanisms and the relationship between its efficacy and molecular variation among tumors will help inform doctors’ decisions about individual patient treatment options, which will improve both overall patient outcomes and patient quality of life by decreasing the use of ineffective therapies. Thus, precision medicine aims to identify molecular markers in cancers to predict patients’ responses to different therapies and provide molecular insights into drug mechanisms.

Most research identifying molecular biomarkers of drug efficacy in cancer have been in the field of pharmacogenomics, which researches genome-level changes as potential biomarkers of drug response^1^. However, in vitro studies indicate that gene expression variation accounts for even more variability in drug sensitivity than genomic changes do and may offer better insight into clinical drug efficacy^2^; yet, there have been few systematic efforts to identify gene expression patterns that influence tumors’ drug sensitivity. While some in vitro studies have sought to identify the relationship between gene expression and drug response by studying differential gene expression when cells are exposed to a drug or by linking cell line gene expression profiles and drug sensitivity^3^, most do not consider real patient outcomes. Previous studies incorporating gene expression and patient drug response were limited to specific cancers or drugs^4^, or focused exclusively on genes implicated in drug metabolism^5^.

The Cancer Genome Atlas (TCGA) is a large dataset with multiple types of molecular data from primary tumors before treatment from a range of cancers and corresponding clinical information, including drug exposures and survival data. The RNA-seq dataset from TCGA is an excellent resource for predictive biomarker identification because pre-treatment gene expression provides a snapshot of a tumor’s transcriptional state at diagnosis, when decisions about treatment options are made. Previously, our group manually standardized drug exposure data to identify gene copy number variations related to survival in a drug-specific manner^6,7^. Analyzing the gene expression data in combination with these clinical data is a similarly powerful strategy for identification of biomarkers for drug-specific survival.

In this study, we perform drug-specific survival analyses to identify genes and gene sets whose pre-treatment expression levels are associated with therapeutic response. We grouped patients based on cancer type and drug exposure and identified genes where patients with high and low pre-treatment expression of that gene had significant survival differences after exposure to that drug. We then clustered these genes into sets based on frequency of co-expression among patients in that group. We identified thousands of gene–drug relationships, with which we subsequently queried PubMed to identify previous reports linking them. Here, we present the results of our analysis, which show promise as potential transcriptomic biomarkers with predictive value for therapeutic response.

## Results

### An integrative pipeline for drug-specific survival analysis

To identify drug-specific survival markers based on gene expression, we integrated drug treatment data, survival data, and RNA-seq gene expression data from TCGA. As part of preprocessing, the gene expression values were binarized based on a high/low threshold calculated separately for each of the 60,483 genes across expression values of all samples for which gene expression data were available. We stratified patients by cancer type and drug exposure: for every unique cancer–drug combination, we defined a patient group as all patients with that cancer treated with the given drug. For each group, we used a log-rank test to compare survival outcomes between patients with high vs. low pre-treatment expression of each gene. In this way, we identified genes whose pre-treatment expression was associated with statistically significant survival differences for that cancer–drug patient group.

Next, for each cancer–drug patient group with at least ten significant genes identified, we used a gene clustering algorithm to define sets of these genes that tended to be co-expressed among patients^8^. To test whether each gene set was predictive of drug-specific survival, we used a log-rank test to compare patients in the relevant group expressing a high number of the genes in that set to patients expressing few of the genes. We calculated the threshold number of genes required for the high expression group for each gene set using the percentage of expressed genes in the gene set across patients in that group using the same method used in the binarization step of preprocessing.

To compare our results with current knowledge about molecular interactions with various cancer therapies, we performed gene set enrichment analysis (GSEA) on each identified set of co-expressing genes to look for enrichment of transcription factor (TF) target genes in the set. We also ran a literature search on PubMed programmatically for each gene–drug combination associated with survival identified in the individual gene analysis as well as each drug–TF combination identified in the GSEA of our co-occurring gene sets.

**Figure 1:**
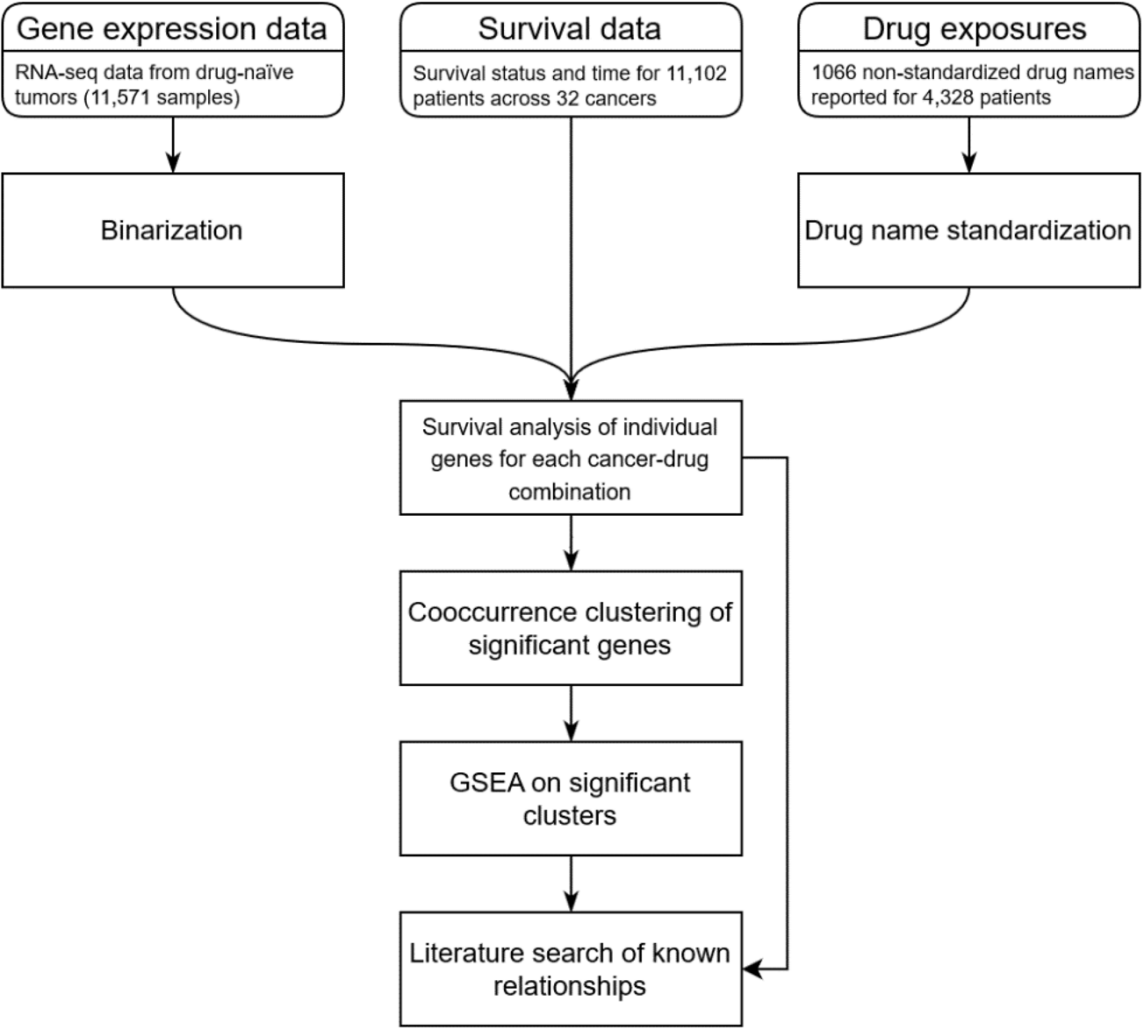
Schematic of the data analysis pipeline used for this study. This figure outlines the major steps of the analysis pipeline.

This pipeline is summarized in Fig. 1. More details on the analysis can be found in the “Methods” section.

### Individual gene expression predictive of drug-specific survival

**Figure 2:**
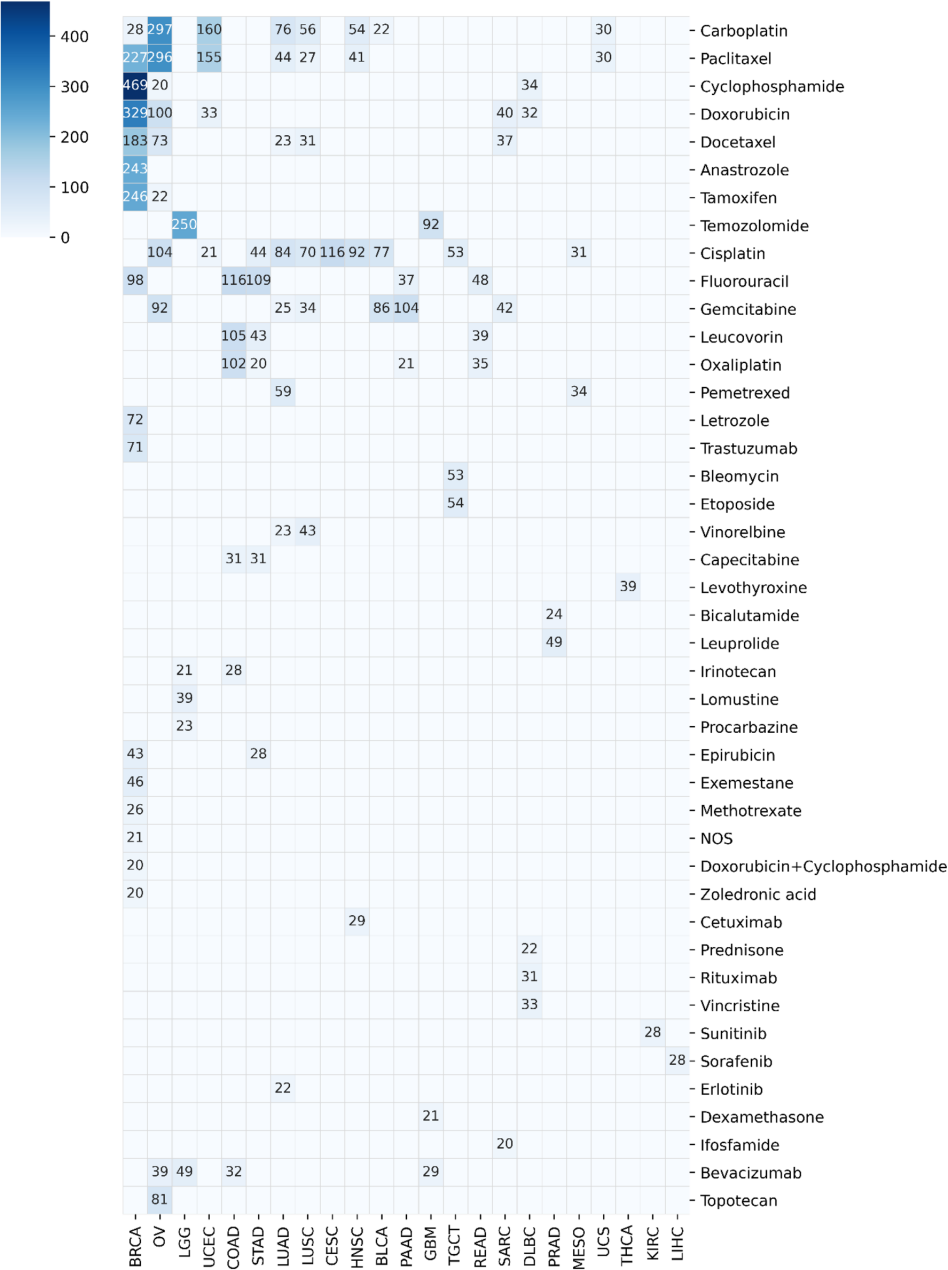
Heatmap of patient numbers by cancer and drug. This heatmap shows the number of patients in each cancer–drug patient group by cancer site and drugs taken. Cancers are listed by TCGA project identifiers, which are defined here: https://gdc.cancer.gov/resources-tcga-users/tcga-code-tables/tcga-study-abbreviations.

TCGA has RNA-seq data for 3533 patients with drug treatment records and survival data. This cohort included 32 cancer types and 284 unique drugs (after drug name standardization). The drug treatment records for these patients consisted of 8836 drug treatment entries, which each included patient information, drug name, time frame of the treatment, etc. After excluding cancer–drug patient groups with fewer than 20 patients, there were 99 groups ranging up to 469 patients. The heat map in Fig. 2 shows the number of patients in each of the 99 cancer–drug groups.

For each cancer–drug patient group, we performed survival analysis on all genes with at least ten low expressors and ten high expressors within the group. We determined significant differential survival using a log-rank test with a 10% false discovery rate (FDR) for the group. Out of 2.2 million cancer–drug–gene combinations tested, we identified 9216 where patients with that cancer who took that drug have significantly different survival rates when stratified by expression of that gene. These occurred across 46 cancer–drug groups, which included 14 cancers and 20 drugs, and we identified 7832 unique genes that were significant in at least one cancer–drug patient group. There were 9212 unique gene–drug interactions identified, with four that were significant in more than one cancer. Table 1 highlights a selected subset of gene–drug interactions we identified, which included the gene–drug interaction that showed the largest difference in drug-specific survival for each of the cancers in our analysis.

**Table 1:** Top gene–drug interactions across cancers.

Cancer	Drug	Gene symbol	p-value	q-value	
BLCA	Cisplatin	RP11-131N11.4	2.28E−06	7.97E−02	
LGG	Temozolomide	RANBP17	1.35E−29	5.29E−25	
BRCA	Doxorubicin	RP11-84A19.4	1.28E−13	5.91E−09	
CESC	Cisplatin	C19orf57	3.29E−08	1.23E−03	
COAD	Fluorouracil	RP11-153F1.1	6.63E−08	2.33E−03	
GBM	Temozolomide	SLC6A6	7.39E−08	2.21E−03	
HNSC	Paclitaxel	ZBTB11	2.56E−08	5.57E−04	
LUAD	Pemetrexed	MDH2	6.52E−09	1.91E−04	
LUSC	Carboplatin	AC096921.2	1.97E−08	5.70E−04	
MESO	Cisplatin	DLC1	1.05E−06	1.55E−02	
OV	Paclitaxel	RP11-60A8.2	7.31E−08	3.38E−03	
SARC	Docetaxel	CMAHP	1.99E−07	4.10E−03	
STAD	Cisplatin	AC024704.2	1.37E−07	3.79E−03	
UCEC	Carboplatin	DDX43P3	2.36E−08	6.15E−04	

This table shows the top gene–drug interactions in each of the 14 cancers in which significant gene–drug interactions were identified. For each cancer–gene–drug combination, the p-value from the log-rank test and the associated q-value (p-values adjusted within each cancer–drug patient group) are shown, along with the Kaplan–Meyer curve illustrating survival differences between patients expressing high levels of the listed gene (orange line) and patients with low expression (blue line).

Our analysis identified many cancer-specific gene–drug interactions, including previously characterized gene–drug interactions as well as ones that are novel and have never been reported in the literature. To gauge the extent of literature support for the identified gene–drug interactions, we queried PubMed for published papers mentioning the drug and gene for each of the 9212 significant gene–drug interactions identified. While most of these gene–drug queries returned no results, indicating that the identified gene–drug interactions have not been previously reported, 531 returned at least one result on PubMed and 158 had three or more papers mentioning the gene and the drug. This strategy identified the gene–drug pairs that are likely to have literature support and helped us confirm multiple examples of our identified gene–drug relationships that have been previously described.

**Figure 3:**
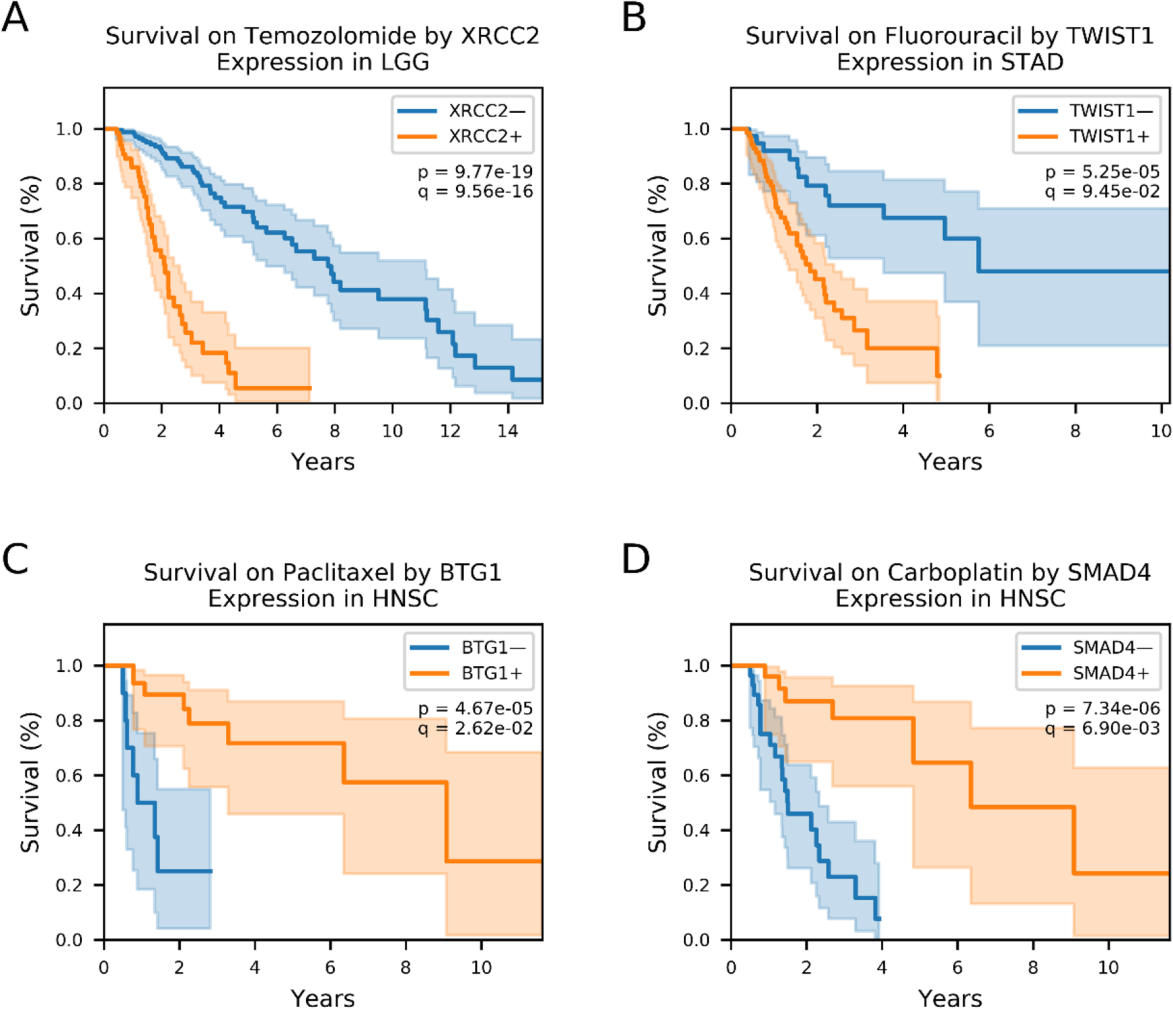
Specific genes are associated with survival following treatment in individual cancer–drug patient groups. Kaplan–Meyer survival curves of patients with the indicated cancer and exposed to the indicated drug, grouped into either high (orange line) or low (blue line) pre-treatment expression levels of the indicated gene. Cancers are referred to their TCGA project identifiers (see Fig. 2). (**A**) Patients who received temozolomide for lower grade glioma, grouped by *XRCC2* expression. (**B**) Stomach adenocarcinoma patients who took fluorouracil, grouped by *TWIST1* expression. (**C**) Patients grouped by *BTG1* expression who took paclitaxel for head and neck squamous cell carcinoma. (**D**) Patients who received carboplatin for head and neck squamous cell carcinoma, grouped by *SMAD4* expression.

One example of a known gene–drug interaction that our analysis identified is between the gene *XRCC2*, a key player in the homologous recombination process, and temozolomide, a methylating agent. In our literature search, we found studies showing that lower *XRCC2* in cancer cells increases temozolomide efficacy by inhibiting their ability to repair the DNA damage induced by temozolomide^9,10^. Our survival analysis showed that lower grade glioma patients with tumors expressing lower levels of *XRCC2* prior to treatment have better outcomes on temozolomide (Fig. 3A), potentially because the function of *XRCC2* counteracts the drug’s mechanism of action. We also identified a previously reported gene–drug relationship between fluorouracil and *TWIST1*. Studies have shown that silencing *TWIST1* can increase certain cancer cells’ sensitivity to fluorouracil^11,12^, which agrees with our findings that, among patients taking fluorouracil for stomach adenocarcinoma, survival outcomes are better for patients with low expression levels of *TWIST1* than for those with high *TWIST1* expression (Fig. 3B).

We also found examples of genes that interact positively with drugs. For example, studies have shown that antiproliferative *BTG1* acts synergistically with paclitaxel in certain cancer cell lines: cells with induced *BTG1* overexpression were more sensitive to paclitaxel and exhibited lower post-treatment expression of chemoresistance genes than controls^13,14^. This aligns with our results, which show that head and neck cancer patients with higher levels of *BTG1* had significantly better survival after taking paclitaxel (Fig. 3C). Additionally, we identified a previously reported relationship between *SMAD4* and carboplatin. Mutations in the *SMAD4* gene have been linked to resistance of platinum-based drugs like carboplatin^15,16^, and our data suggest that head and neck cancer patients on carboplatin stratified by pre-treatment SMAD4 expression have significantly differential survival between the strata (Fig. 3D).

**Figure 4:**
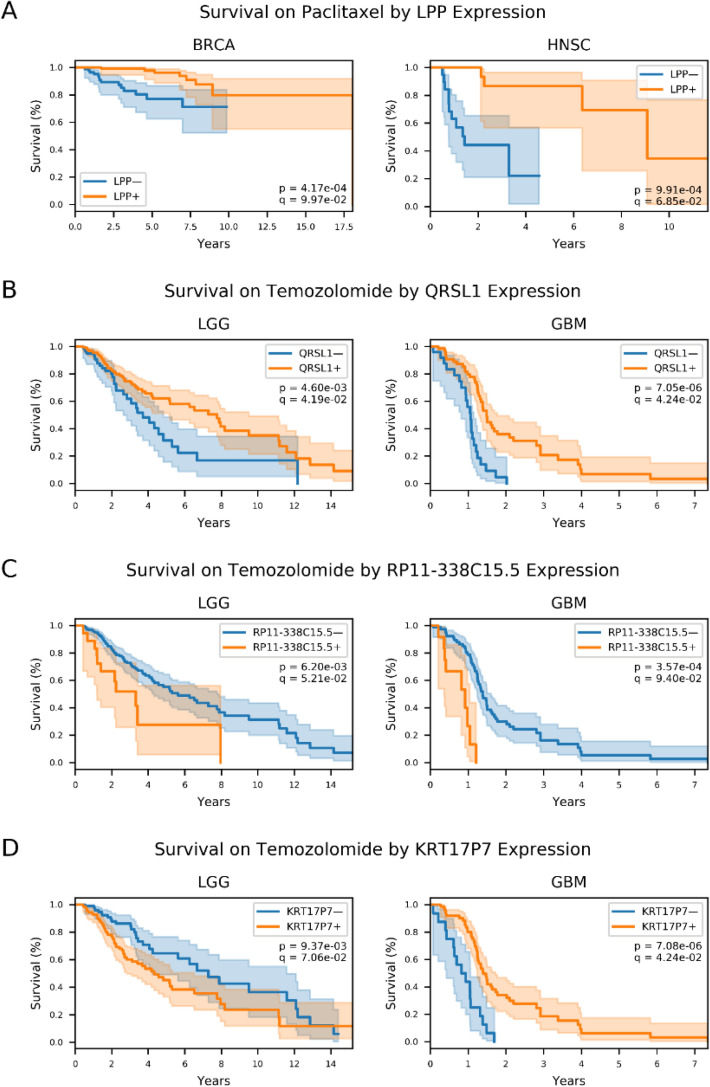
Genes that are associated with drug-specific survival in multiple cancers. Kaplan–Meyer survival curves of patients taking the indicated drug, grouped into either high (orange line) or low (blue line) pre-treatment expression levels of the indicated gene across two different cancers. These are the four gene–drug interactions identified in multiple cancers. (**A**) Survival of breast invasive carcinoma patients (left) and patients with head and neck squamous cell carcinoma (right) who took paclitaxel, grouped by expression of *LPP*. (**B**–**D**) Survival of low-grade glioma patients (left) and glioblastoma (right) patients taking temozolomide, grouped by pre-treatment expression of (**B**) *QRSL1*, (**C**) *RP11-338C15.5*, and (**D**) *KRT17P7*.

We also identified four gene–drug interactions occurring in multiple cancer types. Figure 4 shows the Kaplan–Meyer survival curves comparing high and low expressors of these genes in two different cancer–drug patient groups. Three of the four interactions occurred in low-grade glioma and glioblastoma, while the fourth occurred between *LPP* and paclitaxel in breast invasive carcinoma and head and neck squamous cell carcinoma. A previous study in ovarian tumor-bearing mice linked *LPP* silencing with increased chemosensitivity and improved delivery of paclitaxel to tumor cells, which improved the effectiveness of the drug^17^. In contrast, our analysis found worse patient outcomes in patients with low *LPP* expression; nevertheless, it is encouraging that previous literature has implicated a connection between *LPP* and paclitaxel.

Table 2 summarizes the total numbers of individual genes identified per cancer–drug group and their literature search results. The full list of identified significant gene–drug interactions can be found in Additional file 1. Given the literature support found for many of the identified gene–drug interactions, the novel and highly significant interactions we identified, such as those highlighted in Table 1, are worth investigating for biological insights and validation as biomarkers of drug efficacy.

**Table 2:** Summary of survival analyses of individual genes for each group.

Cancer	Drug	Patients	Genes tested	Significant genes	Gene–drug combinations with ≥ 3 papers	Total gene–drug papers
Bladder urothelial carcinoma	Cisplatin	77	34,921	1	0	0
Brain lower grade glioma	Bevacizumab	49	19,605	623	5	96
	Irinotecan	21	2702	51	1	5
	Lomustine	39	15,412	183	1	198
	Temozolomide	250	39,137	5960	118	2999
Breast invasive carcinoma	Anastrozole	243	43,509	141	0	0
	Cyclophosphamide	469	48,701	52	0	0
	Docetaxel	183	42,009	30	1	8
	Doxorubicin	329	46,315	102	1	4
	Fluorouracil	98	36,614	12	0	0
	Letrozole	72	31,858	16	0	0
	Paclitaxel	227	43,696	184	3	334
	Tamoxifen	246	43,758	43	0	2
Cervical squamous cell carcinoma and endocervical adenocarcinoma	Cisplatin	116	37,450	15	0	3
Colon adenocarcinoma	Fluorouracil	116	35,153	31	0	0
	Leucovorin	105	34,126	22	0	0
	Oxaliplatin	102	33,982	49	1	9
Glioblastoma multiforme	Bevacizumab	29	10,704	1	0	0
	Temozolomide	92	29,925	7	0	0
Head and neck squamous cell carcinoma	Carboplatin	54	27,280	616	12	183
	Cetuximab	29	13,686	451	1	13
	Cisplatin	92	35,953	1	0	0
	Paclitaxel	41	21,738	254	10	107
Lung adenocarcinoma	Carboplatin	76	33,630	12	0	0
	Cisplatin	84	34,231	1	0	0
	Docetaxel	23	7164	2	0	0
	Paclitaxel	44	25,559	6	0	0
	Pemetrexed	59	29,321	191	2	38
Lung squamous cell carcinoma	Carboplatin	56	28,999	7	0	0
	Cisplatin	70	32,214	2	0	0
	Vinorelbine	43	23,941	2	0	0
Mesothelioma	Cisplatin	31	14,732	2	0	1
	Pemetrexed	34	17,203	47	0	1
Ovarian serous cystadenocarcinoma	Carboplatin	297	46,264	4	0	0
	Cisplatin	104	36,549	4	0	0
	Docetaxel	73	32,219	4	0	0
	Doxorubicin	100	36,368	4	0	1
	Gemcitabine	92	35,460	2	0	0
	Paclitaxel	296	46,236	6	0	0
Sarcoma	Docetaxel	37	20,580	3	0	0
	Doxorubicin	40	23,638	2	0	0
	Gemcitabine	42	23,694	17	0	0
Stomach adenocarcinoma	Cisplatin	44	27,671	3	0	0
	Fluorouracil	109	42,242	25	2	30
Uterine corpus endometrial carcinoma	Carboplatin	160	44,684	14	0	0
	Paclitaxel	155	44,321	11	0	0

This table shows the 46 cancer–drug patient groups in which individual genes were identified as significant predictors of survival, along with the number of patients in the group, the number of genes with enough variance across patients in that group to test for differential survival, and the number of genes identified as significant. The last two columns show the number of the significant genes for which the PubMed search found at least three papers along with the total number of papers found supporting the significant genes in that group.

### Gene clusters predictive of drug-specific survival

Clustering the significant genes from each cancer–drug patient group identified 32 different sets of co-expressing genes in eight of the cancer–drug patient groups. For each gene set, we stratified the patients into high and low gene-set expressors based on the percentage of set genes they expressed, and then tested for differential survival between the strata. All identified gene sets showed statistically significant survival differences, and many were more significant than the majority (> 95%) of individual genes in those gene sets. See Additional file 2 for the lists of genes in each gene set.

To elucidate the biological context and meaning of these co-expressing gene sets, we performed gene set enrichment analysis (GSEA) using MSigDB^18^. Of the 32 identified gene sets, 21 were significantly enriched for target genes of at least one transcription factor (TF). We then performed a literature search for each TF–drug combination identified in the GSEA. Table 3 summarizes the gene set analysis results.

**Table 3:** Summary of gene set analysis.

Cancer	Drug	Gene set	Genes in set	Log-rank p-value	Most significant gene p-value	Percent of genes more significant (%)	TFs enriched	TF-drug combinations with 3 + papers	Total TF-drug papers
Brain lower grade glioma	Bevacizumab	A	128	1.94E−09	5.72E−10	**0.8**	0	0	0
		B	175	3.10E−09	1.14E−09	**1.1**	8	0	0
	Temozolomide	M	1619	4.23E−26	1.35E−29	**0.2**	8	1	38
		R	72	5.10E−14	4.62E−14	**1.4**	1	0	1
		S	29	1.00E−13	1.74E−12	**0.0**	0	0	0
		K	49	3.82E−13	1.88E−13	**2.0**	3	0	1
		L	30	1.20E−10	5.48E−15	6.7	9	0	0
		J	106	1.07E−09	3.49E−21	34.0	10	5	24
		N	20	5.50E−08	1.55E−05	**0.0**	0	0	0
		P	56	6.18E−08	3.93E−11	10.7	9	4	90
		F	214	4.43E−07	3.83E−18	17.8	9	0	3
		G	745	6.71E−07	1.43E−13	6.7	8	0	1
		I	11	6.75E−07	2.24E−07	18.2	0	0	0
		Q	89	7.80E−07	1.44E−13	9.0	9	5	59
		D	14	1.10E−06	8.10E−07	7.1	0	0	0
		E	222	5.88E−06	1.63E−09	5.9	7	3	16
		B	171	5.88E−06	1.16E−11	6.4	3	2	7
		A	72	6.91E−06	1.06E−09	**2.8**	8	4	21
		O	44	2.31E−05	1.71E−08	20.5	0	0	0
		H	119	1.54E−04	1.13E−07	15.1	4	0	3
		C	53	3.36E−04	1.82E−09	24.5	0	0	0
Breast invasive carcinoma	Docetaxel	A	14	3.00E−19	1.13E−07	**0.0**	0	0	0
	Tamoxifen	A	19	1.76E−24	1.20E−07	**0.0**	0	0	0
Head and neck squamous cell carcinoma	Carboplatin	B	190	2.52E−06	2.94E−07	**2.6**	8	1	12
		A	136	1.21E−05	1.14E−07	11.0	10	4	22
	Cetuximab	B	84	8.44E−06	1.74E−06	**4.8**	1	1	38
		A	146	7.65E−05	5.08E−07	13.0	10	6	201
		C	71	7.65E−05	9.74E−06	**2.8**	4	2	118
	Paclitaxel	A	16	2.24E−09	2.26E−05	**0.0**	0	0	0
		C	111	3.24E−08	2.56E−08	**0.9**	10	4	46
		B	95	8.37E−07	8.52E−08	**3.2**	7	4	50
Mesothelioma	Pemetrexed	A	14	2.15E−04	2.49E−05	78.6	0	0	0

This table shows the 32 gene sets identified in this analysis, ordered by significance within each group. Columns show the number of genes in the set, the raw (unadjusted) p-values for the log-rank test for that set and for its most significant individual gene, the percent of genes in the set with stronger association with survival than the set (in bold are < 5%, indicating the most useful gene sets), the number of TFs whose target genes were enriched in that gene set, the number of these TFs with at least 3 hits in the PubMed search described, and the total papers found. Letters identifying gene sets (3rd column) represent the order in which the sets were identified during the clustering for that group and match the gene set identification in Additional file 2.

Literature searches revealed that many of these TFs have been discussed in the context of the corresponding drug. For example, we identified a set of genes in head and neck cancer patients taking paclitaxel where patients with high set expression have better survival than low expressors. This gene set was significantly enriched for targets of *NF-κB*, which previous studies found to be related to paclitaxel efficacy^19,20^. Another gene set found in head and neck cancer patients taking carboplatin showing survival differences between high and low expressors was enriched for target genes of *NRF2*, and activation of the *NRF2* pathway has been linked to carboplatin resistance^21,22^. In addition, we identified a set of co-expressed genes enriched for *SRY* targets that exhibits differential survival among low-grade glioma patients on temozolomide, and previous studies have shown a link between the *SRY* pathway and sensitivity to temozolomide^23–25^. This literature support lends credibility to our analysis strategy and findings and suggests that many other TFs identified in our analysis may also contribute to differences in patient response to specific drugs.

## Discussion

Our analysis identified many genes and gene sets whose expression is associated with survival times in various cancer–drug patient groups. With this analysis, we hoped to identify gene–drug interactions that may impact drug efficacy. We found four gene–drug combinations that had a significant association with survival in more than one cancer type. Of these four, three are significant only in closely related cancers: low-grade glioma is a grade II glioma, and glioblastoma multiforme is a grade IV glioma that can arise from a low-grade glioma or develop de novo^26,27^. The low number of gene–drug combinations related to survival that transcended cancer type is likely due to the fact that only a small number of drugs are used in multiple cancers, as illustrated in Fig. 2. In addition, given that most of the gene–drug combinations that are significant in multiple cancers occur in the same tissue, it is possible that many of the identified effects are tissue-specific.

The identified sets of co-expressing genes have several advantages over individual genes as predictors of patient response to therapy. As noted earlier, many of these gene sets stratified the patients into groups with larger survival differences than any of the individual genes in those gene sets. This indicates that, compared to individual genes alone, these gene sets can more accurately separate patients into responders and non-responders. In addition, the gene sets exhibiting the strongest associations with survival contain genes that are part of a similar transcriptome profile and stratify the patients similarly. This means that these gene sets could make better biomarkers than individual genes because they are less vulnerable to measurement errors, minor differences in threshold calculations, or patient-to-patient variability in expression of one or a small number of genes.

While TCGA is a rich resource for genomic and integrative analyses, it is not without its limitations. Drilling down to such fine granularity as cancer- and drug-specific patient groups means that several of the groups contain very few patients. It is likely that we may miss relevant genes due to lower statistical power in smaller cancer–drug patient groups. We mitigated this to the extent possible by excluding small cancer–drug patient groups from our analysis, analyzing only the genes with at least ten low expressors and ten high expressors in a given group, and using a generous FDR to define significance; however, our results would benefit from validation with larger datasets for each cancer–drug patient group. Another limitation is that most of the drug exposure data do not include records of the treatment response, which is why our analysis uses patient survival outcome as an indirect measure of drug efficacy. Since treatment schedules for a given drug can vary between patients and some patients received multiple drugs, patient survival outcome is an imperfect surrogate to measure drug efficacy.

We performed literature searches on PubMed to identify previous reports of the gene–drug and TF–drug relationships identified in our analysis. The results of the PubMed search were reported in Tables 2 and 3. Our PubMed search strategy was rudimentary, and it was not feasible to manually confirm a link between the corresponding gene and drug in the large number of papers from the search. Without a manual review of the papers, the quantity of PubMed search results may not directly represent the amount of literature supporting a particular gene–drug pair. This is especially true when the gene name overlaps with English words, author names, or common abbreviations. However, despite these limitations, our success in manually confirming literature support for multiple examples of gene–drug interactions suggests a high likelihood that literature support exists for many of the gene–drug pairs whose corresponding papers we did not review. Additionally, because it is unlikely that a PubMed query would return no results for a gene–drug pair with a previously reported interaction, we can reasonably conclude that a large majority of our identified gene–drug interactions with no PubMed results are novel and have not been previously reported.

Many of the gene–drug interactions we identified were novel and did not have literature support, and many of our gene sets had no obvious unifying biological interpretation. While many of our identified gene–drug interactions are sufficiently promising to warrant further investigation into the biological mechanisms, these findings could be useful as biomarkers even before the underlying biological mechanism is fully understood. The most significant gene–drug interactions, such as the examples shown in Table 1, have high predictive value, and could serve as biomarkers for drug efficacy.

## Conclusion

In this analysis, we identified many genes that are associated with drug-specific survival outcomes in various cancers. In addition, we were able to identify sets of co-expressed genes that were, in many cases, more strongly associated with patient survival than any of the individual genes in those gene sets, and therefore had higher potential predictive value.

This analysis successfully identified putative biomarkers for drug response in a range of cancers based on gene expression. Therefore, a future research direction is to replicate this analysis in other omics data types available in TCGA, such as DNA methylation, miRNA expression and protein expression, for further insights into variation in drug response among patients. This analysis can then be extended to integrate multiple omics data types for a multi-omics understanding of the molecular variations predictive of drug-specific survival outcomes.

The interactions we identified in our analysis are promising and warrant further investigation, which could yield valuable biological insights into drug mechanisms and variations in drug response. In addition, many of our findings show promise as potential biomarkers of drug response that could be used clinically to predict whether a patient will do well on a drug. Validating these as biomarkers would help doctors in formulating treatment strategies with the highest chances of success for each patient and would be a measurable step toward improving precision medicine.

## Materials and methods

### Data acquisition

We acquired TCGA gene expression data and drug treatment data from the Genomic Data Commons (GDC) database using the GDC Data Transfer Tool. We obtained the file manifest for data files via the GDC API and used the GDC Data Transfer Tool to download files. The parameters used when creating the manifest were “return_type: manifest” along with the filters “files.data_type: Gene Expression Quantification” and “analysis.workflow_type: HTSeq—FPKM-UQ” for RNA-seq data and “files.data_type: Clinical Supplement” and “files.data_format: BCR Biotab” for clinical data.

Patient survival and other clinical data were queried through the GDC API for the most current information.

### Data preprocessing

Drug names from TCGA were standardized based on a manually curated list created by our group previously^7^. The RNA-seq dataset was obtained as FPKM-UQ values and log-transformed for better distribution. We then calculated a binarization threshold to delineate high vs. low expression values for each gene by adapting the StepMiner method described previously^28^. Briefly, gene expression values are ordered from lowest to highest and then fitted with a step function that minimizes the mean square error within the two groups. We tested 400 thresholds for each gene, 200 between evenly distributed bins of samples and 200 evenly distributed through the range of the expression values.

### Survival analysis

All patients with a given cancer and exposed to a given drug were split into high and low expression groups for each gene, and survival was compared between these two groups using a log-rank test. All cancer–drug–gene combinations were analyzed that had a minimum of ten patients in the low and high expression groups. Log-rank calculations and Kaplan–Meyer curves were generated using the lifelines Python package. Q-values were calculated using the Benjamini–Hochberg procedure to control for multiple hypothesis testing with 10% FDR (performed using the fdrcorrection function in the statsmodels Python package).

### Co-occurrence clustering

We adapted a clustering method developed for the analysis of single cell RNA-seq data called co-occurrence clustering to identify sets of co-expressed genes^8^. Briefly, this algorithm constructs a gene–gene graph based on a chi-square pairwise association measure and uses the Louvain algorithm for community detection to identify gene clusters from the graph, then clusters patients similarly based on their expression levels of each gene cluster. This process then iterates for each patient cluster identified. We used this algorithm to identify co-occurring gene sets among the individual genes with significantly differential survival in each cancer–drug patient group. For each gene set identified, we used the percentages of the member genes that were highly expressed for each patient to calculate a binarization threshold to stratify patients into high and low gene-set expression groups and tested for differential survival.

### Literature search

Literature searches were conducted using a Python script with the Bio.Entrez package from Biopython. Queries were formulated as the drug name and the gene or TF name separated by “AND” and relevant PMIDs were retrieved using efetch.

### TF target gene enrichment analysis

To examine whether the gene sets we identified through co-occurrence clustering of drug-specific survival marker genes were significantly enriched for TF targets, we performed gene set enrichment analysis (GSEA) using the Molecular Signatures Database 7.0 (MSigDB). Specifically, we used the GSEA tool (version 4.0.0) to compute overlaps between the gene sets we identified and the sub-collection of MSigDB gene sets that were known or predicted targets of various transcription factors. In MSigDB, the target gene set of a transcription factor is defined as either genes whose predicted binding site for the given TF is within − 1000 to + 500 bp of the transcription start site or genes with upstream cis-regulatory motifs in the promoter region. A detailed explanation can be found at https://www.gsea-msigdb.org/gsea/msigdb/collection_details.jsp#GTRD. We then identified the TFs whose target genes were significantly enriched in each gene set using a 5% FDR to determine significance.

## Supplementary Information

Supplementary Information 1.

Supplementary Information 2.

Supplementary Information 3.

## Data Availability

The TCGA dataset analyzed in this study is available in the GDC repository, https://portal.gdc.cancer.gov/repository. The MSigDB gene sets are available at https://www.gsea-msigdb.org.

## Abbreviations

TCGA: The Cancer Genome Atlas
GSEA: Gene set enrichment analysis
TF: Transcription factor
FDR: False discovery rate
GDC: Genomic Data Commons
