# Supplementary material for: Identifying gene expression patterns associated with drug-specific survival in cancer patients
Source: Sci Rep. 2021 Mar 2;11:5004. doi: 10.1038/s41598-021-84211-y (PMC7925648; doi:10.1038/s41598-021-84211-y)
Supplement: Supplementary file 1 — Supplementary Information 1. [file 41598_2021_84211_MOESM1_ESM.docx]

Additional files

Additional file 1: csv file. Cancer-specific gene-drug interactions. All identified gene-drug interactions by cancer.

Additional file 2: csv file. Genes in each gene set. A list of all genes in each identified gene set.
